# Changes in Kinematics and Muscle Activity With Increasing Velocity During Underwater Undulatory Swimming

**DOI:** 10.3389/fspor.2022.829618

**Published:** 2022-04-15

**Authors:** Keisuke Kobayashi Yamakawa, Hirofumi Shimojo, Hideki Takagi, Yasuo Sengoku

**Affiliations:** ^1^Department of Sport Wellness Sciences, Japan Women's College of Physical Education, Tokyo, Japan; ^2^Department of Health and Sports, Niigata University of Health and Welfare, Niigata, Japan; ^3^Faculty of Health and Sport Sciences, University of Tsukuba, Tsukuba, Japan

**Keywords:** competitive swimming, start and turn, dolphin kicking, 3D motion analysis, EMG, water flume

## Abstract

This study aimed to investigate the changes in kinematics and muscle activity with increasing swimming velocity during underwater undulatory swimming (UUS). In a water flume, 8 male national-level swimmers performed three UUS trials at 70, 80, and 90% of their maximum swimming velocity (70, 80, and 90%V, respectively). A motion capture system was used for three-dimensional kinematic analysis, and surface electromyography (EMG) data were collected from eight muscles in the gluteal region and lower limbs. The results indicated that kick frequency, vertical toe velocity, and angular velocity increased with increasing UUS velocity, whereas kick length and kick amplitude decreased. Furthermore, the symmetry of the peak toe velocity improved at 90%V. The integrated EMG values of the rectus femoris, biceps femoris, gluteus maximus, gluteus medius, tibialis anterior, and gastrocnemius were higher at 90%V than at the lower flow speeds, and the sum of integrated EMGs increased with increasing UUS velocity. These results suggest that an increase in the intensity of muscle activity in the lower limbs contributed to an increase in kick frequency. Furthermore, muscle activity of the biceps femoris and gastrocnemius commenced slightly earlier with increasing UUS velocity, which may be related to improving kick symmetry. In conclusion, this study suggests the following main findings: 1) changes in not only kick frequency but also in kicking velocity are important for increasing UUS velocity, 2) the intensity of specific muscle activity increases with increasing UUS velocity, and 3) kick symmetry is related to changes in UUS velocity, and improvements in kick symmetry may be caused by changes in the muscle activity patterns.

## Introduction

Underwater undulatory swimming (UUS), also known as dolphin kicking or butterfly kicking, is an underwater propelling technique that is used in competitive swimming. During UUS, swimmers propel themselves using undulatory body movements to minimize water resistance by taking a streamlined position with their arms outstretched and held together over their heads. In addition, during UUS, swimmers can avoid the effect of wave drag, which is an additional drag depending on the swimming depth (Lyttle et al., 2000). Therefore, UUS is the quickest form of human locomotion in water and is much faster than surface swimming.

Current international rules permit swimmers to perform UUS for a maximum of 15 m after a start dive and turn, except in breaststroke events. As the highest velocity is achieved immediately after leaving the block or pushing off the wall at the start and turning segments (Takeda et al., 2009; Puel et al., 2012), UUS is performed to minimize deceleration. Previous race analysis studies have reported that a longer underwater distance is related to a faster 15-m total start time (Cossor and Mason, 2001) and that the total time at the start or turning segments is strongly correlated with the overall race performance as well as the time of free-swimming (Mason and Cossor, 2000). Therefore, improvements in UUS could have an important impact on overall race performance (Veiga et al., 2016).

Similar to other swimming strokes, the horizontal swimming velocity during UUS is determined by the product of kick frequency (Hz = cycle/s) and kick length (m/cycle). In UUS, kick length is determined by the horizontal displacement per kick, and kick amplitude (m) is determined by the vertical displacement of the toe or ankle during a one-kick cycle. Previous studies have shown that kick frequency is more related to UUS velocity than length or amplitude (Arellano et al., 2002; Cohen et al., 2012; Houel et al., 2013; Shimojo et al., 2014a; Yamakawa et al., 2017). Several previous studies have indicated that faster vertical toe velocity and angular velocity (e.g., hip extension velocity, hip external rotation velocity, knee extension velocity, knee flexion velocity, and ankle plantar flexion velocity) are also associated with better UUS performance (Atkison et al., 2014; Connaboy et al., 2016; Higgs et al., 2017; Yamakawa et al., 2018). Furthermore, one UUS study reported that the downward toe velocity/upward toe velocity ratio was negatively correlated with the horizontal center of mass velocity and that kick symmetry is also important for UUS performance (Atkison et al., 2014).

In a previous study on front crawl swimming, changes in stroking parameters within the swimming lap were observed (Seifert et al., 2007). In recent years, underwater distances traveled during UUS have considerably increased (Veiga et al., 2014a,b). Considering that underwater distances range between 8 and 15 m for elite swimmers, changes in kicking parameters can probably occur during underwater segments. Therefore, swimmers and coaches need to understand the typical pattern of changes in UUS movements with changing swimming velocity.

A deeper understanding of UUS can be achieved by examining changes in muscular activity, as was previously done during surface swimming. Rouard et al. (1992) reported that the intensity of muscle activity in the upper arm during front crawl swimming increases non-linearly with increasing swimming velocity. Olstad et al. (2017) investigated muscle activity in the upper and lower limbs during breaststroke swimming at 60, 80, and 100% effort and reported that the mean activation pattern remained similar across the different effort levels, but the muscles showed longer activation periods relative to the stroke cycle and increased the intensity of muscle activity with increasing effort. Matsuda et al. (2016) investigated muscle activity in the rectus and biceps femoris during flutter kicking and reported that the intensity of thigh muscles increased with increasing swimming velocity, but that the co-activation level between the muscles did not change. Thus, the intensity of muscle activity in the areas related to specific swimming motions increased with increasing swimming velocity. As mentioned above, several UUS studies have reported that fast angular velocities in hip extension, hip external rotation, knee extension, knee flexion, and ankle plantar flexion are related to high UUS velocity. If these parameters contribute to increasing UUS velocity, the intensity of the related muscle activity (i.e., the activity of the quadriceps femoris, biceps femoris, gluteal muscles, and gastrocnemius) would likely increase with increasing swimming velocity. However, no study has investigated the changes in muscle activity that might occur with increasing UUS velocity.

Therefore, this study aimed to examine the changes in kinematics and muscle activity that occur with increasing swimming velocity during UUS. We hypothesized that with increasing swimming velocity, 1) the kick frequency, vertical toe velocity, and angular velocity increase, 2) kick symmetry improves, and 3) the intensity of muscle activity in the quadriceps femoris, biceps femoris, and gluteal muscles increases.

## Materials and Methods

### Participants

This study included 8 male national-level competitive swimmers (age, 21.1 ± 1.0 years; height, 1.75 ± 0.06 m; and weight, 71.9 ± 7.2 kg), namely, three freestyle swimmers, one backstroke swimmer, one breaststroke swimmer, two butterfly stroke swimmers, and one individual medley swimmer. The mean Fédération Internationale de Natation point score of their personal best times in their specific stroke event was 800.4 ± 81.4 points. All participants had the experience of performing UUS during their daily training. The participants were informed of the risks, benefits, and stresses of the study, and their consent was obtained. This study was approved by the university's research Ethics Committee.

### Experimental Protocol

The experiment consisted of two sessions. In the first session, all participants performed two trials of 25-m UUS at their maximum effort in a 50-m indoor pool. The water temperature was 27.0–28.0°C. The purpose of the first session was to determine the maximum UUS velocity (100%V) that the swimmer could maintain stably, excluding the effect of the push-off start technique, as described by Takeda et al. (2009). The participants had a 30-min free warm-up period before the experiment. During the maximum UUS trials, an examiner walked to match the pace of the swimmer and measured the times at which the swimmer's head passed the 15 and 25 m markers using a manual stopwatch. In an additional experiment, we compared the time measured using the method described above with the time calculated using a video filmed by cameras fixed at the 15 and 25 m points to evaluate the validity of the methodology. The results confirmed that the validity was high because the standard error was ~0.01 s. The average swimming velocity during a 10-m length of the faster trial was calculated as 100%V.

In the second session, the participants performed three UUS trials in a water flume (Igarashi Industrial Works Co. Ltd.; water temperature: 27.0–28.0°C). The standard error of the three-dimensional (3D) velocity distribution in the measurement area was <3% of the set speed. The flow speeds were set to 70, 80, and 90%V of 100%V (70, 80, and 90%V, respectively). In this study, 90%V was determined as the highest flow speed since it was confirmed in a preliminary experiment that swimmers could not complete the desired tasks for testing in the flume at a velocity higher than 90%V. In this study, the mean 70%V was 1.11 ± 0.08 m/s, 80%V was 1.27 ± 0.09 m/s, and 90%V was 1.43 ± 0.10 m/s. The participants had a 30-min free warm-up period before the experiment. In this session, the participants were instructed to swim using UUS at a water depth of 1.0 m as described by Lyttle et al. (2000), and within the same region of the water flume. Therefore, a familiarization session was set up between the warm-up and the experimental task, and the participants confirmed their desired space within the water flume to swim using UUS for motion analysis. Each participant performed this activity until they had completed 10 cycles continuously in a stable position at each flow speed.

### Data Collection and Procedure

**Figure 1:**
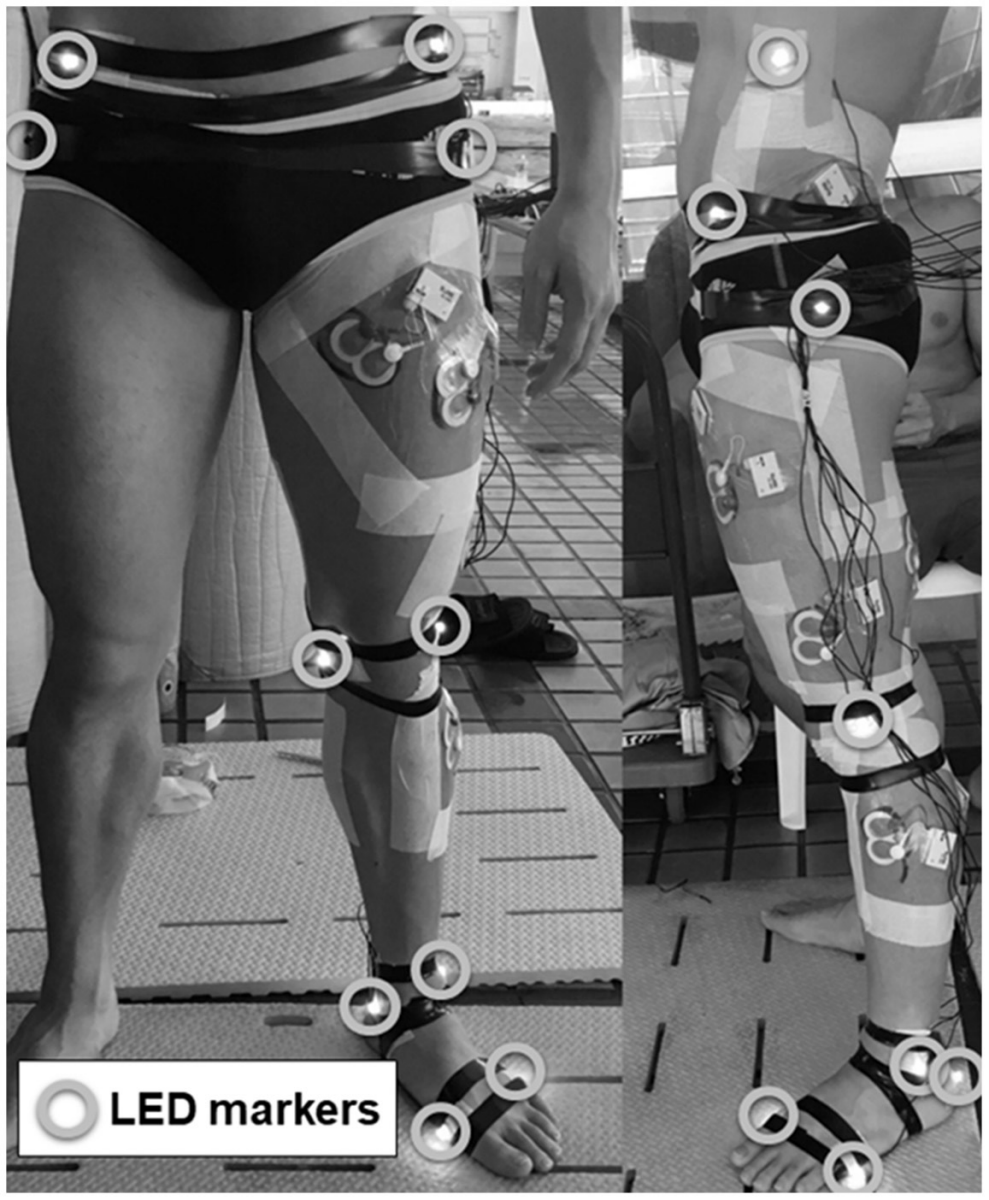
Images of a swimmer's left lower limb with active LED markers attached to 13 anatomical landmarks and surface EMG devices attached to eight muscles. Left: front view; right: lateral view.

**Figure 2:**
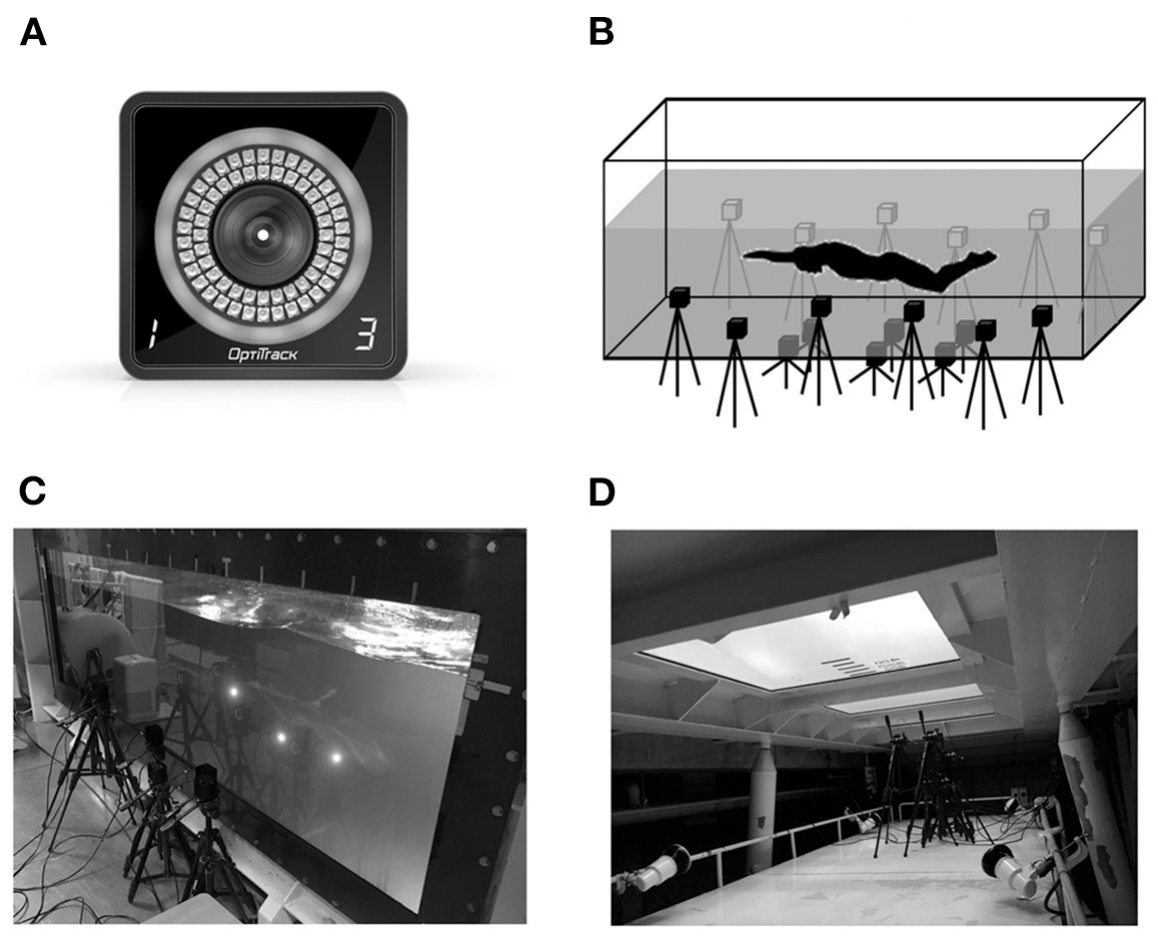
Cameras and experimental settings. **(A)** A camera of the motion capture system. **(B)** The camera setting in the water flume. **(C)** The cameras at the side underwater window of the water flume. **(D)** The cameras at the bottom underwater window of the water flume.

In the second session, we analyzed only the left lower limb movements under the assumption that the movement of both legs was symmetrical during UUS, and LED markers were attached to the participants at 13 body points (Figure 1). The marking points were the right and left 10th ribs at the midaxillary line (“Rib”), right and left hip greater trochanters, right and left anterior superior iliac spine (ASIS), left lateral and medial epicondyles of the femur (Knee_L/Knee_M), left lateral and medial malleoli of the ankle (Ankle_M/Ankle_L), left epiphysis of the first metatarsal (Toe_L), left epiphysis of the fifth metatarsal (Toe_M), and left calcaneal tuberosity (“Heel”). To minimize the effects of the cables used for the LED markers on the swimmer's motion, the cables were fixed with plastic tape along the swimmer's body and bundled onto the swimmer's back. The 3D coordinates during the three trials were acquired using a 3D motion capture system (VENUS-3D, Nobby Tech Inc., Tokyo, Japan; Figure 2A). As shown in Figures 2B–D, 18 cameras were set up adjacent to underwater windows positioned to the side of and below the water flume. The sampling rate of the cameras was set at 100 Hz. To measure 3D space, the origin of the global coordinate system was set at the center of the flume. Flow direction was defined as the direction of the X-axis; the X–Z plane was horizontal to the water surface, and the X–Y plane was vertical to the water surface. The standard error of the 3D coordinates in dynamic calibration was 1.14 mm.

Surface electromyography (EMG) data were collected using a waterproofed telemetric system [DL-5000; input impedance >200 MΩ; Common-mode rejection ratio (CMRR) >110 dB; gain: 400; high cut filter: 1,000 Hz (−3 dB); SandME Inc., Tokyo, Japan; Figure 1], and the data receiver systems included memory storage. The EMG data were measured at a sampling frequency of 1,000 Hz using a 16-bit analog-to-digital conversion, and eight muscles were selected: the left rectus femoris, left vastus lateralis, left adductor longus, left biceps femoris, left gluteus maximus, left gluteus medius, left tibialis anterior, and left gastrocnemius. EMG signals were recorded from the left side of the body using bipolar (interelectrode distance of 0.02 m) disposable Ag-AgCl circular electrodes (Blue Sensor P-00-S, Ambu Inc., Ballerup, Denmark). According to the recommendations of the SENIAM project and Cram et al. (1998), the electrodes were placed as follows: rectus femoris, at the midpoint of the line connecting the anterior superior spina iliaca to the superior part of the patella; vastus lateralis, at two-thirds of the line connecting the anterior superior spina iliaca to the lateral side of the patella; adductor longus, on the medial aspect of the thigh in an oblique direction 4 cm from the pubis; biceps femoris, at the midpoint of the line connecting the ischial tuberosity and the lateral epicondyle of the tibia; gluteus maximus, at the midpoint of the line connecting the sacral vertebrae and the greater trochanter; gluteus medius, at the midpoint of the line joining the crista iliaca to the trochanter; tibialis anterior, at one-third of the line connecting the tip of the fibula and the tip of the medial malleolus; and gastrocnemius, on the most prominent bulge of the muscle. Before the electrodes were affixed, the skin surface was shaved, abraded, and cleaned with alcohol. The electrodes were waterproofed by covering them with water-resistant tape using the methodology described by Kobayashi et al. (2017). To synchronize the kinematic and EMG data, a synchronizer (PTS-110, DKH Inc., Japan) was connected to both trigger channels.

### Data Analysis

Kinematic and EMG data collected during four consecutive kick cycles were used for the following analysis. Four cycles were selected from the middle of 10 cycles because the swimmers' motions were not stable during the first and end cycles. For all kinematic and EMG parameters, the mean values were used to minimize the random error due to inter-cycle variation (Connaboy et al., 2010).

**Figure 3:**
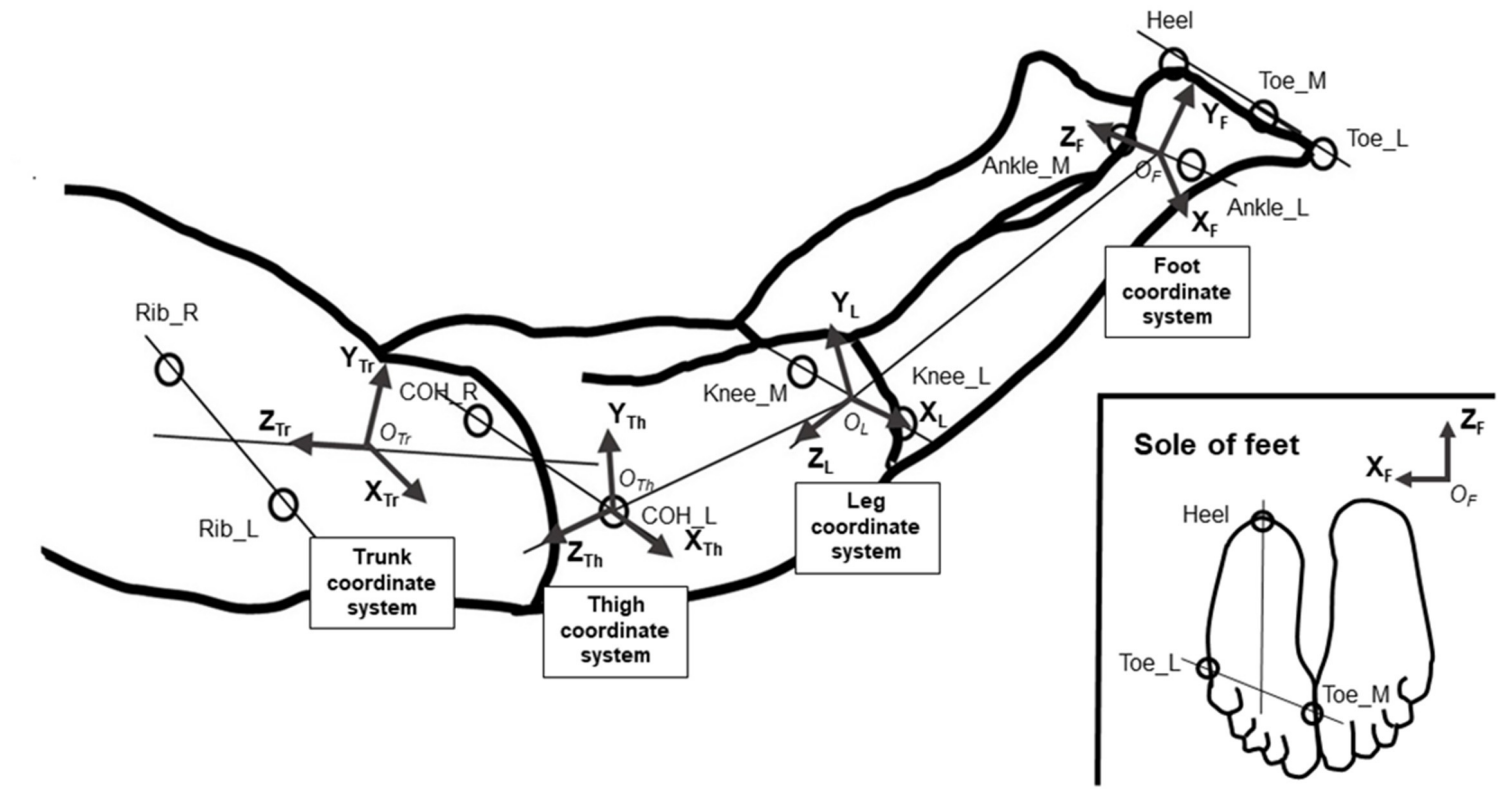
Definitions of the segment coordinate systems.

The coordinates of the right and left centers of the hip joint (COH_R/COH_L) were estimated from the coordinates of the ASIS and the greater trochanter of the hip, in accordance with the recommendations of the Clinical Gait Analysis Forum of Japan (Kurabayashi et al., 2003). For joint angle analysis, the four local coordinate systems in the trunk, thigh, leg, and foot were defined as shown in Figure 3, and the joint angles were calculated as Cardan angles using the four coordinate systems in accordance with Robertson (2004). In the trunk coordinate system, $\vec{X_{Tr}}$ is parallel to a line drawn between COH_R and COH_L, and $\vec{Y_{Tr}}$ is vertical to the plane of the trunk segment (Figure 3). In the thigh coordinate system, $\vec{X_{Th}}$ is parallel to a line drawn between Knee_M and Knee_L, and $\vec{Z_{Th}}$ is parallel to a line drawn between COH_L and the midpoint of Knee_M and Knee_L (Figure 3). In the leg coordinate system, $\vec{X_{L}}$ is parallel to a line drawn between Ankle_M and Ankle_L, and $\vec{Z_{L}}$ is parallel to a line drawn between the midpoint of Knee_M and Knee_L and the midpoint of Ankle_M and Ankle_L (Figure 3). In the foot coordinate system, $\vec{X_{F}}$ is parallel to a line drawn between Toe_M and Toe_L and $\vec{Z_{F}}$ is parallel to a line drawn between the Heel and the midpoint of Toe_M and Toe_L. The origins of the local coordinate systems are designated as *O*_*Tr*_, *O*_*Th*_, *O*_*L*_, and *O*_*F*_ in Figure 3. Using these coordinate systems, the hip joint angle was defined as the angle represented by the trunk and thigh coordinate systems with the origin at the COH_L position; the knee joint angle was defined as the angle represented by the thigh and leg coordinate systems with the origin at the midpoint between Knee_M and Knee_L; and the ankle angle was defined as the angle represented by the leg and foot coordinate systems with the origin at the midpoint between Ankle_M and Ankle_L. At these angles, the rotation around the X-axis was defined as flexion/extension, the rotation around the Y-axis as adduction/abduction, and the rotation around the Z-axis as internal/external rotation. We decided to analyze the hip extension/flexion angle, hip abduction/adduction angle, hip internal/external rotation angle, knee flexion/extension angle, ankle plantar flexion/dorsal flexion angle, and ankle abduction/adduction angle. For analysis, the peak angle, ranges of motion (ROM), and peak angular velocities were calculated. To compare joint movement patterns, joint angle data during a kick cycle were interpolated to 101 percentiles for time normalization, and an individual ensemble curve was created using data from four kick cycles to minimize inter-cycle variation. The mean ensemble curve for all participants was created for each angle using individual ensemble curves.

In this study, the UUS cycle began at the maximum peak of the Z-displacement of the toe (Toe_L) position and ended at the next highest peak, and one UUS cycle was divided into three phases as follows, as reported by Arellano et al. (2002): downward kick (DK), first upward kick (UK-1), and second upward kick (UK-2). The UK-1 and UK-2 phases were separated according to the time at which the horizontal velocity component of Toe_L was greater than the vertical velocity component during upward kicking. To compare the phase structures between the different swimming velocity trials, the relative duration was calculated (as a percentage) and normalized to the cycle duration in each phase. Kick frequency was defined as the reciprocal of the duration of a one-kick cycle. The kick amplitude was defined as the vertical distance between the highest and lowest positions of Toe_L during one UUS cycle using the absolute displacement. Swimming velocity was defined as the sum of the horizontal velocity at the midpoint between the COH and the flow speed, and the average swimming velocity during one UUS cycle was calculated. Kick length was defined as the product of the swimming velocity and the duration of a one-kick cycle. The mean and peak vertical toe velocities during the downward and upward kick phases were calculated from the coordinates of Toe_L. The symmetry between the downward and upward toe velocities was evaluated by dividing the downward values by the upward values, as described by Atkison et al. (2014).

Raw EMG signals were recorded on a computer, and signal processing was conducted using numerical analysis software (MATLAB 2013a, MathWorks Inc., USA). To remove motion artifacts and prevent aliasing, raw EMG signals were filtered using a band-pass filter (20–500 Hz). The filtered EMG signals were rectified and smoothed using a low-pass filter (15 Hz, fourth-order Butterworth). To compare muscle activity patterns, the EMG amplitude was normalized to the mean value for the UUS cycle in the 70%V trial, as described by Turpin et al. (2011). The normalized EMG data were interpolated to 101 percentiles for time normalization, and an individual ensemble curve during the UUS cycle was created using the data of four kick cycles. The mean ensemble curve for all participants was created for each muscle using individual ensemble curves. To evaluate the quantitative value of the muscle activity, the integrated EMG signal (iEMG) was calculated for a one-kick cycle. The sum of the iEMG signals during the cycle (sum iEMG) was calculated as the total muscle activity in the left lower limb.

### Statistical Analysis

All parameters are reported as mean and standard deviation (mean ± SD). Statistical processing was conducted using the bell curve in Excel (SSRI Inc., Japan). To compare the data between trials, the normality of all data was confirmed using the Shapiro-Wilk test, and sphericity was checked using the Mauchly sphericity test. When the data showed normal distribution, the variables were compared between each trial using repeated-measures analysis of variance (ANOVA), and Bonferroni *post-hoc* corrections were performed to test differences between trials. Effect sizes (as partial eta-squared values) for ANOVA were used to interpret meaningful effects (Knudson, 2009). When data distribution was not normal, the variables were compared between each trial using the Friedman test, and Bonferroni *post-hoc* corrections using a Wilcoxon signed-rank test were performed to test differences between trials. In these statistical tests, the statistical significance level (*a*) was set at 0.05.

## Results

Table 1 shows the results of kinematic analyses. As shown by the ANOVA and Friedman test, there was a significant main effect of velocity on kick frequency (*p* < 0.01, ES = 0.58), kick length (*p* < 0.01, ES = 0.22), kick amplitude (*p* < 0.01, ES = 0.26), mean downward toe velocity (*p* < 0.01, ES = 0.57), peak downward toe velocity (*p* < 0.01, ES = 0.39), mean upward toe velocity (*p* < 0.01, ES = 0.39), peak upward toe velocity (*p* < 0.01, ES = 0.39), and symmetry of peak toe velocity (*p* < 0.01, ES = 0.12). The results of the *post-hoc* tests showed that kick frequency increased with increasing swimming velocity (all *p* < 0.05), whereas kick length decreased with increasing swimming velocity (all *p* < 0.05). Kick amplitude was lower in the 90%V trial than in the 70 and 80%V trials (both *p* < 0.05). Mean downward toe velocity, mean upward toe velocity, and peak upward toe velocity increased with increasing swimming velocity (all *p* < 0.05). The peak downward toe velocity was higher in the 90%V trial than in the 70 and 80%V trials (both *p* < 0.05). The symmetry of peak toe velocity was higher in the 90%V trial than in the 70%V trial (*p* < 0.05).

**Table 1:** Results of kinematic variables in the 70, 80, and 90%V trials.

**Variable**	**Unit**	**70%V**	**80%V**	**90%V**	***P*-Value**	**ES**
Kick frequency	(Hz)	1.46 ± 0.18	1.75 ± 0.26	2.11 ± 0.33	<0.01^a,b,c^	0.58
Kick length	(m/cycle)	0.77 ± 0.06	0.72 ± 0.09	0.68 ± 0.08	<0.01^a,b,c^	0.22
Kick amplitude	(m)	0.60 ± 0.03	0.58 ± 0.05	0.54 ± 0.05	<0.01^b,c^	0.26
DK phase	(%)	46.1 ± 3.7	45.4 ± 2.8	46.3 ± 2.9	0.40	0.02
UK-1phase	(%)	38.0 ± 4.1	39.0 ± 3.2	39.5 ± 3.0	0.88	NP
UK-2 phase	(%)	18.7 ± 3.0	19.0 ± 2.5	18.3 ± 2.1	0.38	0.02
Mean downward toe velocity	(m/s)	1.81 ± 0.21	2.00 ± 0.19	2.31 ± 0.19	<0.01^a,b,c^	0.57
Peak downward toe velocity	(m/s)	3.59 ± 0.27	3.76 ± 0.32	4.07 ± 0.19	<0.01^b,c^	0.39
Mean upward toe velocity	(m/s)	1.54 ± 0.20	1.72 ± 0.23	1.92 ± 0.19	<0.01^a,b,c^	0.39
Peak upward toe velocity	(m/s)	2.56 ± 0.31	2.83 ± 0.38	3.16 ± 0.28	<0.01^a,b,c^	0.39
Symmetry of mean toe velocity	(a.u.)	1.18 ± 0.08	1.17 ± 0.10	1.20 ± 0.08	0.49	0.03
Symmetry of peak toe velocity	(a.u.)	1.41 ± 0.14	1.35 ± 0.18	1.29 ± 0.09	0.04^b^	0.12

a *Significantly different between 70 and 80%V trials (P < 0.05)*;

b *Significantly different between 70 and 90%V trials (P < 0.05)*;

c *Significantly different between 80 and 90%V trials (P < 0.05); ES, effect size; NP, tested using a non-parametric test*.

**Figure 4:**
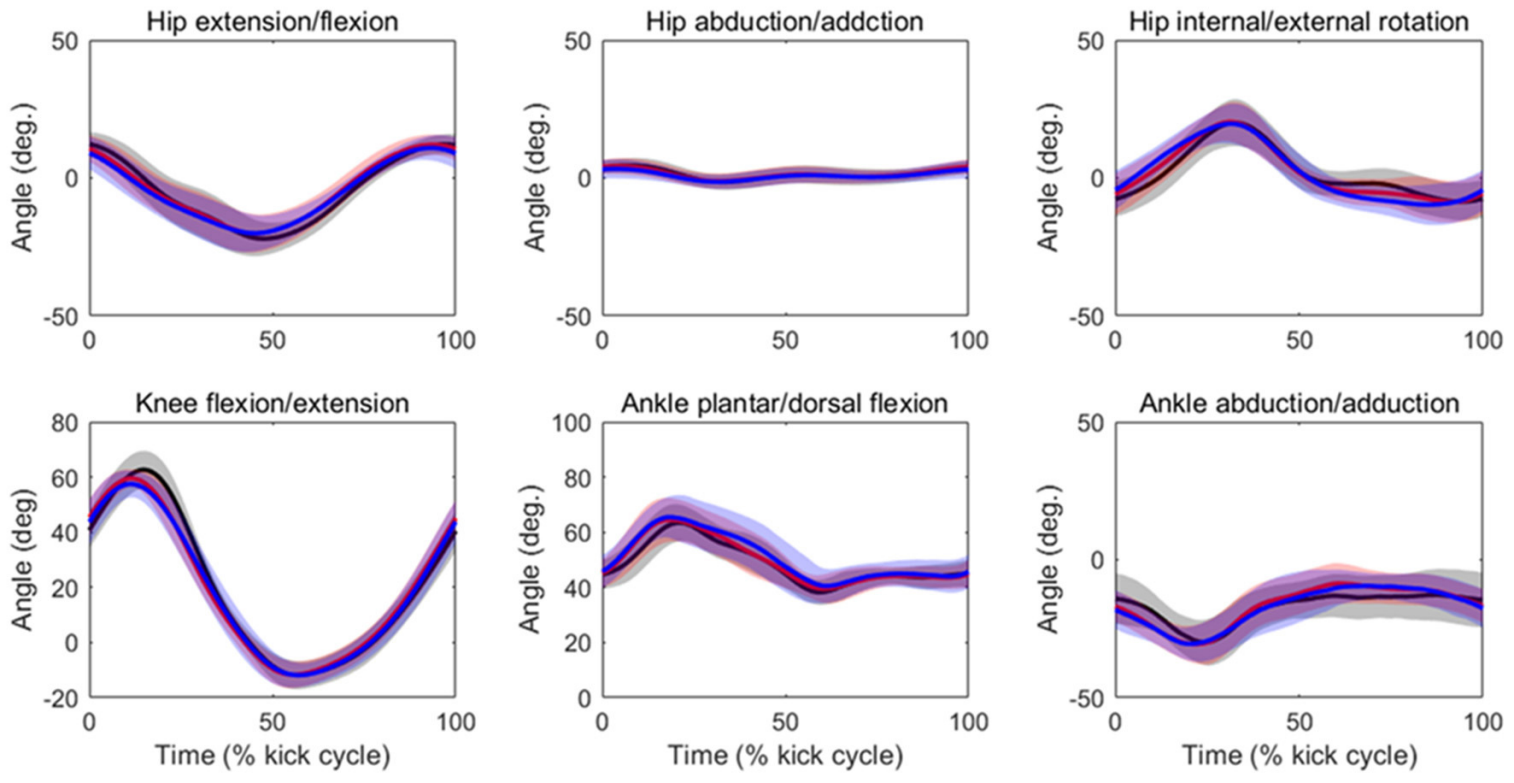
Mean patterns and standard deviations for the hip, knee, and ankle joint angle data in the 70%V (black), 80%V (red), and 90%V (blue) trials.

Table 2 summarizes the analyses of peak joint angles, ROM, and peak joint angular velocities. The results of the ANOVA and Friedman test indicated that there was a significant main effect of velocity in the peak hip extension angle (*p* = 0.04, ES = 0.03), peak hip flexion angle (*p* = 0.03, ES = 0.02), hip flexion/extension ROM (*p* = 0.01, ES = 0.08), peak knee flexion angle (*p* = 0.03, ES = 0.19), knee flexion/extension ROM (*p* = 0.04, ES = 0.12), peak ankle plantar flexion angle (*p* = 0.01, ES = 0.08), peak hip extension velocity (*p* < 0.01, ES = 0.13), peak hip flexion velocity (*p* < 0.01, ES = 0.09), peak hip internal rotation velocity (*p* < 0.01, ES = 0.32), peak hip external velocity (*p* < 0.01, ES = 0.20), peak knee flexion velocity (*p* < 0.01, ES = 0.40), peak knee extension velocity (*p* < 0.01, ES = 0.33), peak ankle plantar flexion velocity (*p* < 0.01, ES = 0.22), and peak ankle dorsal flexion velocity (*p* < 0.01, ES = 0.25). The *post-hoc* tests indicated that the hip flexion/extension ROM, peak knee flexion angle, and knee flexion/extension ROM were lower at 90%V than at 70%V (all *p* < 0.05). The peak ankle plantar flexion angle was higher at 90%V than at 70%V (*p* < 0.05). Peak hip extension velocity and peak knee flexion velocity increased with increasing swimming velocity (all *p* < 0.05). The peak hip flexion velocity, peak hip external rotation velocity, peak knee extension velocity, and peak ankle dorsiflexion velocity were higher in the 90%V trial than in the 70 and 80%V trials (all *p* < 0.05). The peak hip internal rotation velocity and peak ankle plantar flexion velocity were higher in the 90%V trial than in the 70%V trial (both *p* < 0.05). Figure 4 shows the mean patterns of the hip, knee, and ankle joint angle data in the 70, 80, and 90%V trials.

**Table 2:** Summary of peak joint angle, range of motion (ROM), and peak joint angular velocity in the 70, 80, and 90%V trials.

**Variable**	**Unit**	**70%V**	**80%V**	**90%V**	***P*-Value**	**ES**
Peak hip extension angle	(deg.)	12.9 ± 4.2	12.9 ± 4.2	11.4 ± 4.8	0.04	0.03
Peak hip flexion angle	(deg.)	23.1 ± 6.9	20.9 ± 7.7	20.9 ± 7.7	0.03	0.02
Hip flexion/extension ROM	(deg.)	36.0 ± 4.7	33.8 ± 6.1	32.3 ± 6.8	0.01^b^	0.08
Peak knee flexion angle	(deg.)	63.7 ± 6.9	61.0 ± 3.7	58.7 ± 4.8	0.03^b^	0.19
Knee flexion/extension ROM	(deg.)	76.2 ± 7.7	73.1 ± 6.5	71.5 ± 4.5	0.04^b^	0.12
Peak ankle plantar flexion angle	(deg.)	63.8 ± 7.4	65.1 ± 7.8	66.2 ± 9.0	0.01^b^	0.08
Peak hip extension velocity	(deg./s)	174.3 ± 41.5	194.6 ± 49.8	215.5 ± 47.5	<0.01^a,b,c^	0.13
Peak hip flexion velocity	(deg./s)	181.5 ± 34.6	188.2 ± 44.2	210.2 ± 47.1	<0.01^b,c^	0.09
Peak hip internal rotation velocity	(deg./s)	181.9 ± 56.0	206.2 ± 32.6	251.1 ± 42.8	<0.01^b^	0.32
Peak hip external rotation velocity	(deg./s)	219.1 ± 68.9	242.1 ± 78.5	309.3 ± 98.7	<0.01^b,c^	0.20
Peak knee flexion velocity	(deg./s)	333.2 ± 76.2	409.0 ± 97.7^b^	498.4 ± 90.6	<0.01^a,b,c^	0.40
Peak knee extension velocity	(deg./s)	446.6 ± 39.8	454.6 ± 62.6	526.1 ± 57.8	<0.01^b,c^	0.33
Peak ankle plantar flexion velocity	(deg./s)	239.3 ± 52.3	300.7 ± 106.3	354.1 ± 113.4	<0.01^b^	0.22
Peak ankle dorsal flexion velocity	(deg./s)	185.4 ± 34.0	209.0 ± 66.9	279.3 ± 103.0	<0.01^b,c^	0.25

a *Significantly different between 70 and 80%V trials (P < 0.05)*;

b *Significantly different between 70 and 90%V trials (P < 0.05)*;

c *Significantly different between 80 and 90%V trials (P < 0.05); ES, effect size*.

**Figure 5:**
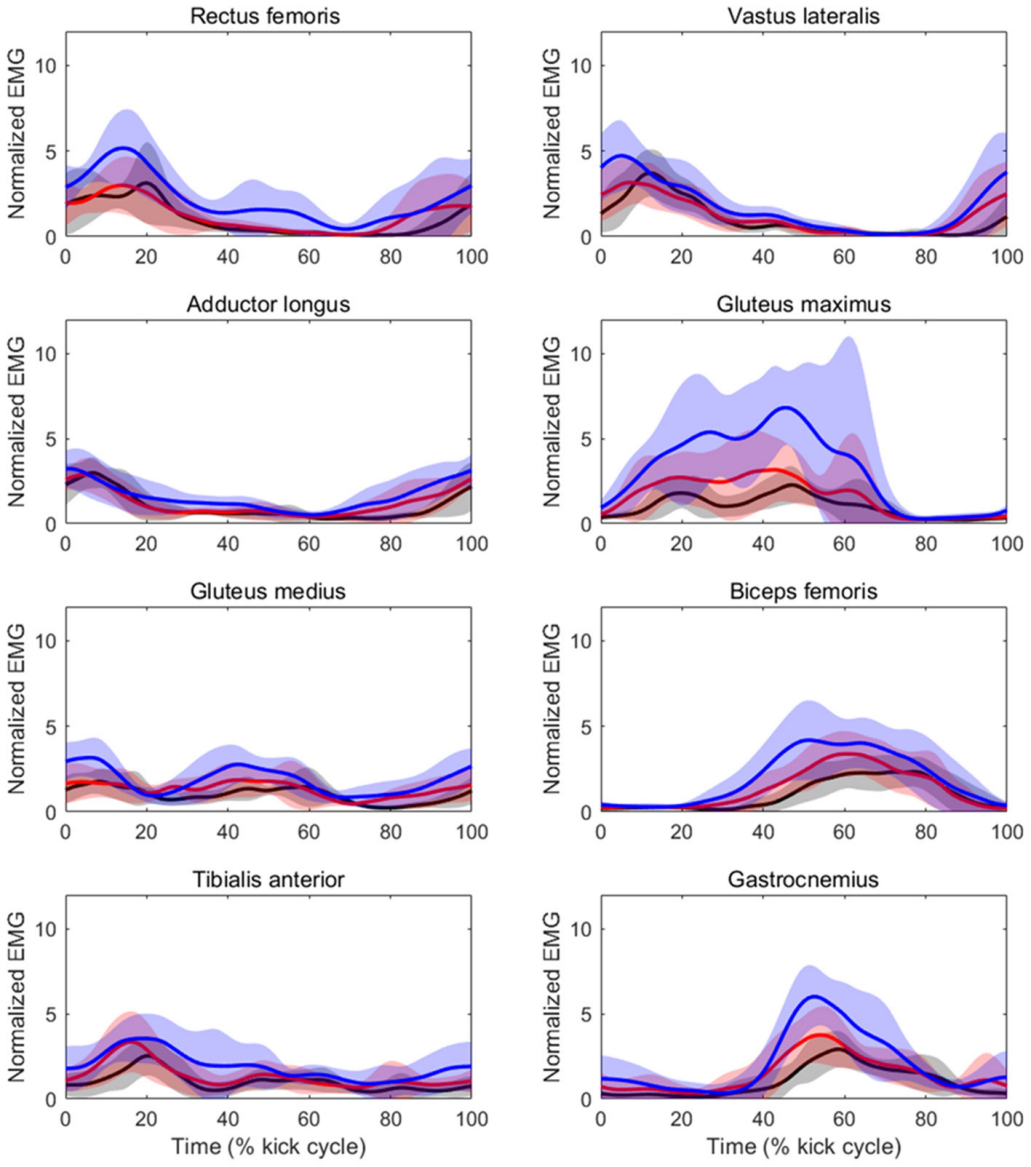
Mean patterns and standard deviations for the EMG envelopes normalized to the mean of the 70%V trial at 70%V (black) and 80%V (red).

Table 3 shows the results of iEMG for each muscle as well as the sum iEMG. The ANOVA and Friedman test revealed a significant main effect of velocity in the iEMGs of the rectus femoris (*p* < 0.01, ES = 0.41), gluteus maximus (*p* < 0.01, ES = 0.37), gluteus medius (*p* < 0.01, ES = 0.04), biceps femoris (*p* < 0.01, ES = 0.12), tibialis anterior (*p* < 0.01, ES = 0.08), gastrocnemius (*p* < 0.01, ES = 0.15), and sum iEMG (*p* < 0.01, ES = 0.41), except for those of the vastus lateralis and adductor longus. The *post-hoc* tests showed that the iEMGs of the rectus femoris, gluteus maximus, gluteus medius, and tibialis anterior were higher in the 90%V trial than in the 70 and 80%V trials (all *p* < 0.05). The iEMGs of the biceps femoris and gastrocnemius were higher in the 90%V trial than in the 70%V trial (both *p* < 0.05). The sum iEMG increased with increasing swimming velocity (all *p* < 0.05). Table 4 shows the changes (%) in the iEMG from 70%V. Figure 5 shows the mean patterns for the EMG envelopes normalized to the mean of the 70%V trial in the 70, 80, and 90%V trials.

**Table 3:** Results of iEMG for each muscle and sum iEMG in the 70, 80, and 90%V trials.

**Variable**	**Muscle**	**Unit**	**70%V**	**80%V**	**90%V**	***P*-Value**	**ES**
iEMG	Rectus femoris	(mV·s)	58 ± 14	63 ± 17	86 ± 20	<0.01^b,c^	0.41
iEMG	Vastus lateralis	(mV·s)	90 ± 16	95 ± 26	108 ± 36	0.13	0.10
iEMG	Adductor longus	(mV·s)	70 ± 62	65 ± 48	75 ± 63	0.07	NP
iEMG	Gluteus maximus	(mV·s)	20 ± 9	29 ± 17	44 ± 19	<0.01^b,c^	0.37
iEMG	Gluteus medius	(mV·s)	43 ± 23	46 ± 23	53 ± 23	<0.01^b,c^	0.04
iEMG	Biceps femoris	(mV·s)	67 ± 22	80 ± 31	90 ± 30	0.01^b^	0.12
iEMG	Tibialis anterior	(mV·s)	39 ± 17	43 ± 16	52 ± 23	<0.01^b,c^	0.08
iEMG	Gastrocnemius	(mV·s)	97 ± 25	117 ± 46	133 ± 48	0.01^b^	0.15
iEMG	Sum of muscles	(mV·s)	484 ± 83	538 ± 93	639 ± 86	<0.01^a,b,c^	0.41

a *Significantly different between 70 and 80%V trials (P < 0.05)*;

b *Significantly different between 70 and 90%V trials (P < 0.05)*;

c *Significantly different between 80 and 90%V trials (P < 0.05); ES, effect size; NP, tested using a non-parametric test*.

**Table 4:** Magnitudes of changes (%) in iEMG from 70%V and from 80%V.

**Variable**	**Muscle**	**Unit**	**70–80%V**	**70–90%V**	**80–90%V**
Change of iEMG	Rectus femoris	(%)	9.2 ± 12.7	51.1 ± 29.5	40.8 ± 38.8
Change of iEMG	Vastus lateralis	(%)	4.2 ± 18.3	18.9 ± 33.2	13.0 ± 16.0
Change of iEMG	Adductor longus	(%)	1.7 ± 11.5	7.6 ± 11.3	7.1 ± 16.7
Change of iEMG	Gluteus maximus	(%)	34.3 ± 27.8	124.3 ± 74.4	73.8 ± 65.0
Change of iEMG	Gluteus medius	(%)	10.5 ± 13.0	29.2 ± 19.7	16.9 ± 11.2
Change of iEMG	Biceps femoris	(%)	18.9 ± 18.1	39.1 ± 38.9	17.7 ± 31.2
Change of iEMG	Tibialis anterior	(%)	13.8 ± 16.9	34.2 ± 18.1	18.5 ± 9.2
Change of iEMG	Gastrocnemius	(%)	18.2 ± 20.1	36.5 ± 30.8	16.0 ± 22.0
Change of iEMG	Sum of muscles	(%)	11.7 ± 9.1	33.0 ± 10.7	19.6 ± 12.0

## Discussion

### Kinematics

Our results showed that kick frequency increased with increasing UUS velocity, while the kick length decreased, and that the ES of kick frequency was the highest among all kinematic variables. In UUS, kicking frequency is the main parameter that influences UUS performance (Connaboy et al., 2009). Cohen et al. (2012) used simulation to investigate whether increasing kick frequency during UUS affects the streamwise forces on the tethered swimmer, and their simulation showed that the mean streamwise forces on the tethered swimmer increased linearly with increasing kick frequency. Accordingly, the thrust during UUS may increase with increasing kick frequency if the swimming motion does not change. However, in this study, kick length decreased with an increase in kick frequency. This suggests that the swimmers increased their kick frequency, sacrificing their propulsive ability to increase their UUS velocity.

The increase in kick frequency can be explained by changes in kick amplitude, vertical toe velocity, and joint angular velocity. Kick amplitude decreased in the 90%V trial, and the ROM of hip flexion-extension and knee flexion-extension also decreased in the 90%V trial. These results suggest that the decrease in kick amplitude owing to the decrease in ROM contributes to the increase in kick frequency. Although a small amplitude in an undulatory movement can contribute to a reduction in drag (Hochstein and Blickhan, 2014; Pacholak et al., 2014), it does not lead to an increase in thrust production. In contrast, an increase in vertical toe velocity not only contributes to an increase in kick frequency but is also related to vortex generation and thrust production (Ungerechts et al., 2000). Therefore, swimmers should increase vertical toe velocity rather than reduce kick amplitude to increase UUS velocity.

In this study, both mean vertical toe velocities in the downward and upward kick phases increased with increasing UUS velocity. Furthermore, the peak hip extension velocity and peak knee flexion velocity increased with increasing swimming velocity. These results support our hypothesis that the vertical toe velocity and angular velocity increase with increasing UUS velocity. Higgs et al. (2017) indicated that an increase in hip extension velocity contributes to an increase in vertical toe velocity during upward kicking and that an increase in knee flexion velocity contributes to a reduction in the relative duration of the deceleration phase, such as the UK-2 phase (Arellano et al., 2002). However, in this study, the relative duration of UK-2 did not change across the different UUS velocities. Therefore, we speculate that the increase in knee flexion velocity contributed to the increase in the upward toe velocity.

The peak hip internal/external rotation velocity was faster in the 90%V trial than in the other trials. Shimojo et al. (2019) indicated that the external rotation of the foot during downward kicking helps vortex generation in the sole of the foot and may contribute to an increase in propulsion. As shown in Figure 4, the hip joint rotated internally in the first half of the DK phase and rotated externally in its latter half, and the joint movement pattern did not change across different UUS velocities. Therefore, the external rotation velocity of the foot in the 90%V trial may have increased upon increasing the hip external rotation velocity. Although this study did not measure propulsion, our results support the notion that external rotation of the foot is related to increased UUS velocity.

Previous hydrodynamic UUS studies have indicated that efficient swimmers might obtain more propulsion during upward kicking than inefficient swimmers (Arellano et al., 2002; Hochstein and Blickhan, 2011), although the main propulsion of UUS was observed during downward kicking. Atkison et al. (2014) reported that the symmetry of the vertical toe velocity was correlated with UUS velocity and that the peak toe velocity had a higher correlation coefficient than the mean toe velocity. To explain this observation, the authors reported that vortex shedding during the UUS cycle seemed to appear depending on the timing of the peak toe velocity. Therefore, this study suggests that an improvement in the symmetry of peak toe velocity is related to an increase in UUS velocity. Based on the results of the present and previous studies, we propose that the symmetry of the peak toe velocity is a variable related not only to higher UUS performance in swimmers but also to an increase in UUS velocity.

### Muscle Activation

Table 3 shows that the sum iEMG, which indicates the total muscle activity in the left lower limb, increased with increasing UUS velocity. Yamakawa et al. (2017) reported that, in the UUS, the intensity of muscle activity in the rectus abdominis, rectus femoris, biceps femoris, tibialis anterior, and gastrocnemius increased upon increasing kick frequency. In this study, kick frequency increased with increasing UUS velocity. Therefore, our results support the view that the swimmers increased their swimming velocity by increasing kick frequency, which was achieved by increasing the intensity of muscle activity.

The iEMGs of the rectus femoris at 90%V were enhanced compared with those at 70 and 80%V. Furthermore, the ES of the rectus femoris was the highest among all muscles. This result was expected. However, the iEMGs of the vastus lateralis and adductor longus did not change across the different UUS velocities, although these muscles are involved in knee extension and hip flexion. This may be because the standard deviations of the iEMGs in the vastus lateralis and adductor longus were higher than those in the rectus femoris. This suggests that the changes in the intensity of muscle activity of the vastus lateralis and adductor longus involved larger differences across individuals compared with that of the rectus femoris.

The iEMG values of the biceps femoris and gastrocnemius at 90%V were higher than those at 70%V. It was observed that activity within these muscles began slightly earlier at higher swimming velocities (as shown in Figure 5). The functions of the biceps femoris are hip extension and knee flexion, and those of the gastrocnemius are ankle plantar flexion and knee flexion. These results suggest that, with increasing swimming velocity, swimmers changed the intensity and start time of muscle activity for breaking the knee extension and ankle dorsiflexion quicker and for starting the hip extension and ankle plantar flexion earlier, resulting in an improvement in the symmetry of the vertical toe velocity.

The iEMG of the gluteus maximus at 90%V was enhanced compared with that at other velocities, and the magnitude of the increase was the highest among the eight muscles (Table 4). The timing of activation matched the start time of the hip external rotation and hip extension (Figures 4, 5). The main functions of the gluteus maximus include hip extension and external hip rotation. Therefore, we speculate that the swimmers increased the intensity of gluteus maximus activity to rotate the hip joint externally more quickly as well as to extend the hip joint more quickly to increase UUS velocity.

The iEMG of the gluteus medius at 90%V was increased compared with those at other velocities, but the ES was the lowest among all muscles. Although the gluteus medius is a strong hip abductor, distinct hip abductive movements through the UUS cycle were not observed (Figure 4). In an anatomical atlas (Schünke et al., 2006), it was noted that the anterior part of the gluteus medius acting alone helped to flex and internally rotate the hip joint, whereas the posterior part of the gluteus medius acting alone helped to extend and externally rotate the hip joint. In this study, the gluteus medius activity had two peaks during a cycle, and the timing of activation matched the start time of hip flexion, internal rotation, and extension (as shown in Figures 4, 5). However, EMG signals of the gluteus medius were collected from the middle fibers. Therefore, it was difficult to determine how the increase in gluteus medius activity contributed to the change in the kinematics.

The iEMG of the tibialis anterior at 90%V was higher than those at other velocities. The peak of tibialis anterior activity appeared during the DK phase across different UUS velocities (Figure 5). The main function of the tibialis anterior is ankle dorsiflexion. Connaboy et al. (2016) indicated that ankle dorsal flexion velocity is a factor that contributes to maximal UUS velocity. Therefore, fast ankle dorsiflexion is important for achieving higher maximal UUS performance. Furthermore, an increase in ankle dorsiflexion velocity can contribute to an increase in downward toe velocity. From these findings, our results suggest that the increase in tibialis anterior activity may contribute to increasing downward toe velocity, increasing the maximal UUS velocity.

### Practical Implications

Our kinematic results indicate that not only does the kick frequency contribute to an increase in UUS velocity, but that the kick length, kick amplitude, vertical toe velocity, angular velocity, and kick symmetry also change with an increase in UUS velocity. Shimojo et al. (2014a) reported that swimmers could not increase their UUS velocity by reducing kick length, kick amplitude, and Froude efficiency when they were required to immediately increase their kick frequency. Accordingly, it can be speculated that swimmers should not focus only on kick frequency to increase their UUS velocity. Our results emphasize that swimmers should increase the vertical toe velocity and/or angular velocity rather than kick frequency to increase UUS velocity because these changes are important for increasing thrust during UUS.

The results of the muscle activity recordings suggest that the intensity of muscle activity of the rectus femoris, gluteus maximus, gluteus medius, biceps femoris, tibialis anterior, and gastrocnemius muscles increased with increasing UUS velocity. In particular, gluteus maximus activity increased by approximately 120% when swimming velocity increased by 20%. Thus, the load on the gluteus maximus may be very high compared with that on other muscles when a swimmer trains at a high intensity using UUS. If muscle fatigue occurs at the gluteus maximus, it is difficult for swimmers to increase the hip external rotation velocity and hip extension velocity during UUS. Therefore, we recommend that swimmers train the gluteus maximus to maintain a higher UUS performance.

Furthermore, the results of the muscle activity pattern suggest that early initiation of muscle activity in the biceps femoris and gastrocnemius contributes to an improvement in kick symmetry. Therefore, swimmers should ensure that they activate the biceps femoris and gastrocnemius earlier to improve kick symmetry, resulting in increased UUS velocity.

### Limitations

As these experiments were conducted in a water flume, the conditions differed from those of a race where swimming is performed in relatively static water. For instance, the kick amplitude during UUS has been reported to be higher in a water flume than in static water because swimmers try to stay in one place (Shimojo et al., 2014b). However, we were able to accurately change the swimming velocity using a water flume. The added drag associated with wearing LED markers and wireless EMG devices might affect swimming performance. Passive drag increases when 3D markers are worn (Kjendlie and Olstad, 2012), which may compromise swimming performance (Washino et al., 2019). Therefore, we speculated that our participants could not maintain 100%V using UUS in the water flume because of the added drag. Furthermore, this study had several other limitations, including one-leg evaluation, differences from a 100% assessment in a swimming pool followed by evaluations in the swimming pool, small sample size, and the inclusion of swimmers with different main swimming strokes.

## Conclusion

This study investigated the changes in kinematics and muscle activity with increasing swimming velocity during UUS. Our kinematic results indicate that the swimmers increased kick frequency and decreased kick length with increasing swimming velocity, and that the increases in kick frequency were caused by increases in the vertical toe velocity and joint angular velocity, and by a decrease in kick amplitude. At the highest swimming velocity, internal, and external rotation velocities of the hip increased. Changes in the hip rotational velocity may have affected the external rotation of the foot, resulting in an increase in thrust during the DK phase. These results suggest that the changes in not only the kick frequency but also in the kicking velocity are important for increasing the UUS velocity. In addition, the results indicate that the improvement in the symmetry of the peak toe velocity was related to an increase in UUS velocity. The results of muscle activity recordings indicated that the total muscle activity in the lower limbs increased with increasing UUS velocity, especially those of the rectus femoris, gluteus maximus, gluteus medius, biceps femoris, tibialis anterior, and gastrocnemius, which were at the highest levels at the highest swimming velocity. Furthermore, we observed that muscle activity in the biceps femoris and gastrocnemius began slightly earlier with increasing UUS velocity, which may be related to improving kick symmetry. These findings provide insights into improvements in UUS performance and appropriate velocity control strategies for swimmers and coaches.

## Data Availability Statement

The datasets generated for this study are available on request to the corresponding author.

## Ethics Statement

The studies involving human participants were reviewed and approved by Japan Women's College of Physical Education. The patients/participants provided their written informed consent to participate in this study. Written informed consent was obtained from the individual(s) for the publication of any potentially identifiable images or data included in this article.

## Author Contributions

KY created the main conceptual ideas for the paper. All authors contributed to the manuscript writing.

## Funding

This study was supported by Grant-in-Aid for Young Scientists (B) (17K13136) from Japan Society for the Promotion of Science (JSPS), and a cooperative research grant of advanced research initiative for human high performance (ARIHHP), University of Tsukuba.

## Conflict of Interest

The authors declare that the research was conducted in the absence of any commercial or financial relationships that could be construed as a potential conflict of interest.

## Publisher's Note

All claims expressed in this article are solely those of the authors and do not necessarily represent those of their affiliated organizations, or those of the publisher, the editors and the reviewers. Any product that may be evaluated in this article, or claim that may be made by its manufacturer, is not guaranteed or endorsed by the publisher.
